# Evaluating the impact of integrated care clinics on Asian American populations’ mental health

**DOI:** 10.3389/fmed.2026.1686739

**Published:** 2026-05-21

**Authors:** Sammi Wong, Christopher Wan, Steven Sust

**Affiliations:** 1 Department of Psychiatry, OBH One Brooklyn Health Brookdale Hospital Medical Center, New York, NY, United States; 2 Department of Psychiatry, Mount Sinai Hospital, New York, NY, United States; 3 Department of Psychiatry, Stanford University, Stanford, CA, United States

**Keywords:** Asian American, collaborative care, consultation, depression, ethnic minority, immigrant, integrated care, stereotype

## Abstract

There has been a persistent underutilization of mental health (MH) services among Asian Americans. Many researchers have attempted to explore possible etiologies and to find more effective solutions for the considerable ethnic disparities in MH. Several studies have found that integrated psychiatry and community health primary care increases treatment engagement and access to specialty MH services for low-income Asian Americans. As a result, integrated care models may serve as a crucial preventative model for proper MH care within this population. By evaluating the data from a primary care clinic, Northeast Medical Services (NEMS), which utilizes the integrated care model to serve a predominantly Asian American population, we hope to determine the impact interprofessional collaboration between primary care physicians and psychiatrists has on patients’ MH outcomes. Consequently, this helps inform our understanding of help-seeking pathways that could serve Asian American immigrants in the United States.

## Introduction

A majority of individuals seek mental health (MH) care from primary care institutions. Since the 1970’s, integrated care has garnered increasing attention as a possible solution to deliver more comprehensive and patient-centered care (1). Of note, integrated care models are the overarching term that encompasses Collaborative Care Model (follows the University of Washington (UW) AIMS model), interprofessional care, co-located care, and the consultation model. Under the Washington Collaborative Care Model (AIMS CoCM), psychiatrists work with the Behavioral Health Care Manager, review the registry of patients, and make recommendations that are shared with the primary care provider (PCP) (2). Under the consultation model, the PCP consults directly with an MH provider, and the MH provider bills the payer directly for professional-to-professional services on behalf of the patients the PCP sought advice for (2). The integrated care clinic discussed in this article follows a combination of both the Collaborative Care Model and the consultation model. PCPs within the clinic seek consultation from the only MH provider affiliated, and there is no formal registry; outcome metrics rely on the clinic’s EMR system.

Although these clinics attempt to increase MH care access, there continues to be a persistent underutilization among specific populations, such as Asian Americans in the United States. Many researchers have attempted to explore possible etiologies and to find more effective solutions for the considerable ethnic disparities in MH. One study found that integrated psychiatry and community health primary care increases treatment engagement and access to specialty MH services for low-income Chinese Americans (3). Kwong et al. (4) found that among 57 participants enrolled in a collaborative care model for low-income Chinese-Americans with clinical depression, there was a reported significant reduction of depressive symptoms and improved MH functioning from baseline to 4-month follow-up assessments. Within a coordinated care model in association with a statewide collaborative care program, Ratzliff et al. (5) found that the rate of improvement for depression was slightly higher for Asians in a culturally sensitive clinic than for Asians in other clinics, 35% vs. 24%, respectively. Bao et al. (6) analyzed the randomized controlled trial: Prevention of Suicide in Primary Care Elderly: Collaborative Trial (PROSPECT) and found that under this intervention, which utilized collaborative depression care management, patients with no college education demonstrated a progressively smaller decline in depression symptoms than college-educated patients, specifically, the minority population saw a slower decline in the first 4 months. These studies indicate that many institutions have aimed to improve MH care through integrated care models, as these models may prevent multiple missed opportunities of offering evidence-based MH care to Asian Americans. However, there have not been any recent studies published on outcome metrics from primary care clinics that serve predominantly Asian American populations. Thus, we aim to bridge this knowledge gap.

Based on the pilot data we obtained from North East Medical Services (NEMS), a primary care clinic that serves a predominantly uninsured, low-income Asian American population, we hope to determine if the interprofessional collaboration between primary care physicians and psychiatrists that exists within NEMS had an impact on patient outcomes, specifically the depression symptoms in relation to the PHQ-9 scores.

## Methods

### Study setting

NEMS was founded in 1968 in San Francisco’s Chinatown. This non-profit community health center has since expanded to include over two dozen health centers in the San Francisco Bay Area. Providing both primary care and specialty medical services, NEMS aims to provide affordable, comprehensive, and culturally sensitive care by serving patients of Asian ethnicity, with providers speaking various languages and dialects, including English, Cantonese, Mandarin, Korean, Vietnamese, and more. NEMS is an example of a Federally Qualified Health Center (FQHC), which is a nonprofit community health center serving the San Francisco Bay Area. FQHCs are safety net clinics that provide comprehensive preventative health services to rural and underserved communities. According to the Health Resources and Services Administration, these centers qualify for specific funding and reimbursement from Medicare and Medicaid. NEMS’ mission is to provide affordable, comprehensive, and quality healthcare services in a linguistically competent and culturally sensitive manner.

NEMS follows the model in which the psychiatrist provides informal MH consultation to primary care physicians (PCPs) regarding medications or psychiatric diagnoses via evidence-based brief interventions or telepsychiatry. A PCP consults a psychiatrist directly and seeks professional expertise without the psychiatrist seeing the patient. While there may be multiple PCPs, there is only one psychiatrist at NEMS. Since the electronic medical record system at NEMS does not have its own patient tracker, the behavioral health care manager utilizes a paid subscription for the UW Registry tracker to track and share patient information, thus ensuring continuity of care.

### Study design and data collection

A retrospective cohort study was conducted of patients referred to MH in seven NEMS clinics between May 1, 2019, and November 21, 2020. Data from the following NEMS clinics were queried: Clement, Daly City, Noriega 1400, Noriega 1450, San Bruno, Stockton, and Taraval. Inclusion criteria were patients of any age who were seen by the consulting psychiatrist at least once and had Patient Health Questionnaire-9 (PHQ-9) scores recorded at their first and last visit during the study period.

Demographics (age, gender, and clinic location), number of MH visits, PHQ-9 scores at MH or primary care visits, and whether the patient was taking a psychotropic medication at each visit were collected. PHQ-9 scores were collected as part of the standard routine at MH or primary care visits, rather than at set intervals in the study period. Whether a psychotropic medication was initiated, maintained, or discontinued was measured to provide insight into what psychopharmacological management was completed for patients in the collaborative care model.

A paired *t*-test on patients’ first and last visit PHQ-9 scores, in addition to univariate descriptive statistics, was conducted. All analyses were performed in SAS OnDemand. Average follow-up between first and last PHQ-9 test was 149 days, with a standard deviation (SD) of 137 and a standard error (SE) of 13. The average number of behavioral health encounters was 8.6, median was 6. Dropouts were majority male (54.1%) vs. our study population (27.3%).

## Results

A total of 112 patients met the study inclusion criteria. The mean age was 39.8 years (Minimum = 14, Maximum = 80, SD = 17.3), and the majority (72.3%) of patients were female. There was no appreciable variance in outcomes by gender. Females had an average PHQ-9 decrease of 5.58 (SD: 7.66) vs. males of 5.48 (SD: 4.7460). The two clinics with the most patients (Daly City, Stockton) had 70–75% of participants who were female.

Most patients were seen in the Daly City (49.1%) and Stockton (27.7%) clinics. The number of total MH encounters during the study period was positively skewed (Mean = 8.6, SD = 9.32, Median = 6). During the study period, 44.6% of patients never took a psychotropic medication, while 14.3, 14.3, and 26.8% maintained, started, or discontinued a psychotropic medication, respectively (Table 1).

**Table 1:** Descriptive statistics [*N* (%)].

Patients’ profile	Number of Patients [N (%)]
Total patients*	112
Age - Mean (SD)*	39.8 (17.3)
Female	81 (72.3)
Primary clinic	
Clement	3 (2.7)
Daly City	55 (49.1)
Noriega 1400	7 (6.3)
Noriega 1450	8 (7.1)
San Bruno	6 (5.4)
Stockton	31 (27.7)
Taraval	2 (1.8)
BH encounters - Median (IQR)*	6 (2, 11)
BH medication status	
Never taking	50 (44.6)
Maintained	16 (14.3)
Started	16 (14.3)
Discontinued	30 (26.8)

This table lists descriptive statistics of the 112 patients included in the study.

*Denotes the statistics that do not follow the *N* (%) format and only represent *N*.

The median follow-up time between first and last PHQ-9 was 105 days (IQR 42, 204). The median number of behavioral health encounters was 6 (IQR 2, 11). There was strong statistical evidence (*t*_111_ = −8.446, *p* < 0.001) of a difference between the initial and final PHQ-9 questionnaires, with an average reduction of 5.55 points (SD = 6.96). When assessing the severity of PHQ-9 scores across initial and final questionnaires, there was a decrease in the proportion of severe, moderately severe, and moderate scores, and an increase in the proportion of mild and none/minimal scores (Table 2). These outcomes were similar across the female and male genders.

**Table 2:** PHQ-9 scores [*N* (%)].

Metrics	Initial	Final
Mean (SD)*	13.9 (5.8)	8.4 (5.7)
Median (IQR)*	14.0 (9.5, 18.0)	7.5 (4.0, 11.0)
PHQ-9 severity		
Severe	19 (16.96)	6 (5.36)
Moderately severe	32 (28.57)	8 (7.14)
Moderate	33 (29.46)	31 (27.68)
Mild	23 (20.54)	37 (33.04)
None/minimal	5 (4.46)	30 (26.79)

This table lists the mean and median of the initial and final PHQ-9 scores and lists the number of patients within each PHQ-9 severity rank.

*Denotes the statistics that do not follow the *N* (%) format and only represent *N*.

Patients who did not meet study inclusion criteria, i.e., did not have a follow-up PHQ-9 score, had similar initial PHQ-9 scores (12.8, SD 5.8) as the study population. These patients were predominantly male (54.1%), and the median number of BH encounters was 2 (IQR 1, 3.5).

## Discussion

Our study must be interpreted in light of its limitations, given our single-arm observational study design. With no control group, we cannot establish causal inferences about the implementation of collaborative care at NEMS. Our data is also subject to various confounding biases. Resultingly, the observed downward trend in depression outcomes cannot be definitively attributed to the integrated care model versus patients experiencing the natural course of disease or traditional models of MH care. Other limitations include variance in duration between the initial and final PHQ-9 scores across patients. Depending on the patient, some final PHQ-9 scores were reported in the range of 3–6 months after the initial PHQ-9 scores were reported. There is also attrition bias introduced by respondents who did not complete follow-up PHQ-9 questionnaires (possibly due to common survey fatigue in the Asian American population). Additionally, due to the absence of ethnic demographic information in our data and the high ethnic heterogeneity of Asian Americans, the study results may not generalize across individual ethnicities.

Given known cultural and gender-related factors in mental health stigma and help-seeking, the dominance of female participants in this study may have also influenced outcomes. This gender difference may allude to how Asian American males are resistant to accessing MH resources due to their socialization to masculine norms and Asian cultural values. For example, Kim et al. illustrate how masculine norms exerted both direct and indirect effects on help-seeking intentions, with the effect of face concerns mediated through self-stigma (7). Iwamoto et al. also suggest that adherence to masculine norms and avoidant coping strategies play a role in Asian American men’s decreased usage of MH services (8). This may be reflected in our study, given that patients lost to follow-up were majority male and had fewer encounters with our BH clinics.

There is a statistically distinct reduction in PHQ-9 scores; the mean change of 5.55 points on the PHQ-9 might also be considered clinically meaningful, as depression severity groups are scored in increments of 5 (i.e., 5–9 is mild severity, 10–14 is moderate severity, 15–19 is moderately severe, and 20 + is severe). In our study period, when collaborative care was implemented at NEMS, there was a decrease in the proportion of patients with moderate to severe depression as scored by the PHQ-9.

### Future research design

Integrated care models’ foundational characteristics promote easier access to MH care, especially for the Asian American community and its values that are historically influenced by Confucianism (9). Patients prefer having streamlined services that decrease their number of medical visits; thus, colocation of primary care and MH services increases access to this population. As the PCP, behavioral health care manager, and psychiatrist are in direct communication, healthcare providers take a leading role in monitoring patients’ treatment, which builds patients’ trust and rapport. Additionally, PCPs communicate with patients in their native language which eliminates the need for interpretation services.

Future directions include quality improvement studies identifying facilitators and barriers to the successful implementation of primary care-mental health integration in a multilingual primary care setting. Subsequent studies to address this may include qualitative focus groups with patients of different ethnicities, immigration statuses, and genders, or with stakeholder interviews of providers and staff who work in these clinics. This may increase understanding of resilience factors and help-seeking pathways that primarily serve Asian American immigrants in the United States. By incorporating culturally tailored strategies (e.g., culturally-sensitive and linguistically-aligned mental health questionnaires in destigmatized primary care settings, culturally sensitive workforce supporting completion, interpretation, treatment planning, etc.) and team-based care models in patient-centered community centers, more ethnic minority groups could access the MH services they need. Specifically, these culturally tailored strategies may highlight cultural elements such as family, community, and collectivism, which resonate with Asian Americans and make them feel that these strategies were designed for them. Those who feel an intervention is likely designed for them will participate more fully in it and ultimately benefit from it.

Furthermore, future studies that investigate food insecurity and other social determinants of health could further clarify particular risk factors to monitor such as immigration status, English literacy, or care of multigenerational family dependents that could potentially influence a patient’s physical and mental health. We hope to utilize deep structure tailoring strategies that involve incorporating the cultural, social, historical, environmental, and psychological forces to influence the target health behavior in the proposed target population. For example, this may include incorporating messaging family (with the patient’s consent) as a way to utilize messengers with whom the individual may identify with—through either common ancestral background or shared experiences—to encourage the patient to follow through with certain treatment plans. Evidence suggests that culturally tailored interventions improve disease knowledge, health care access, care coordination, clinical outcomes, and patient satisfaction in racial and ethnic minoritized communities (10, 11).

As we have mentioned previously, there is a high ethnic heterogeneity among the Asian American population; some are immigrants, some are US citizens not born in the US, and some are US citizens born in the US. Thus, in our future data collection, we hope to gather a representative sample across gender and Asian American subgroups. This will help clarify whether integrated care functions similarly across diverse communities and subpopulations. While our data did not specify whether integrated care clinics improve Asian Americans’ mental health, it has showcased data that a potential connection can be made and that collaboration between PCPs and psychiatrists may encourage this population to utilize MH services more.

Our work, overall, serves as an initial exploration into a problem that is currently understudied and not well-understood. Future work would benefit from a more rigorous and systematic study of the many patients served by FQHCs like NEMS to establish the real-world impact and causality of our services. Given the underserved communities in FQHC safety nets, we hope that our findings may facilitate more resources and collaboration to further care for these communities.

## Data Availability

The original contributions presented in the study are included in the article/supplementary material, further inquiries can be directed to the corresponding author.
